# Acquired Dyslexia and Dysgraphia in Chinese

**DOI:** 10.1155/2005/323205

**Published:** 2006-01-09

**Authors:** Wengang Yin, Shengxi He, Brendan Stuart Weekes

**Affiliations:** ^1^Chinese Academy of ScienceBeijingChina; ^2^University of SussexBightonUK

## Abstract

Understanding how the mappings between orthography and phonology in alphabetic languages are learned, represented and processed has been enhanced by the cognitive neuropsychological investigation of patients with acquired reading and writing disorders. During the past decade, this methodology has been extended to understanding reading and writing in Chinese leading to new insights about language processing, dyslexia and dysgraphia. The aim of this paper is to review reports of patients who have acquired dyslexia and acquired dysgraphia in Chinese and describe the functional architecture of the reading and writing system. Our conclusion is that the unique features of Chinese script will determine the symptoms of acquired dyslexia and dysgraphia in Chinese.

## 1. Introduction

Reports of patients with selective impairment to reading and/or writing to dictation have had a substantial impact on the development of cognitive models of reading and writing in English [6,12,29]. The primary example is the dissociation between acquired surface dyslexia and phonological/deep dyslexia. Acquired surface dyslexia refers to impaired oral reading of irregular words in English such as pint accompanied by preserved oral reading of regular words such as *punt* and nonwords such as *zint*. An irregular word will typically be regularised in surface dyslexia e.g. *pint* read as if it rhymed with mint. These errors are legitimate though incorrect alternative readings of the components of an irregular word and thus can be referred to as LARC responses [25]. An impaired ability to read nonwords accompanied by preserved oral reading of irregular and regular words, is called acquired phonological dyslexia. Deep dyslexia is a severe form of phonological dyslexia wherein patients make semantic errors when reading low imageability words e.g. “justice” read as “peace” in addition to poor oral reading of nonwords. Analogous symptoms in writing can be observed in patients who have surface, phonological and deep dysgraphia. These dissociations in reading and writing can be explained by assuming two independent pathways (at least) are used for normal reading and writing and that damage to one or the other pathway results in a pattern of selective disorders of dyslexia and dysgraphia [6,29].

The motivation for the present paper is to review reports of patients who have acquired dyslexia and dysgraphia in Chinese. These reports have lead to the development of a functional architecture of the reading and writing system which is described in detail in the latter part of the paper. Our method is the cognitive neuropsychological approach to investigating reading and writing disorders pioneered by Marshall and Newcombe [19,20] and by colleagues such as Coltheart [4–6], Hillis and Caramazza [11] and Patterson [23–27, 29].

## 2. Chinese languages and script

Chinese languages include Mandarin (Putonghua) which is spoken in Mainland China and Taiwan and Cantonese which is spoken in Hong Kong and Southern China. These languages contain a relatively small number of syllables that can be used in isolation or in combination to represent a single morpheme or multimorphemic word. Chinese syllables are typically made up of an onset that is a single consonant (note that some

ISSN 0953-4180/05/$17.00 © 2005 – IOS Press and the authors. All rights reserved syllables e.g. ai have no consonantal onset) followed by a short or a long vowel that is followed by a coda comprising at least one consonant. The onset of each syllable is invariant however the rime (i.e., the vowel plus the final consonant combination) can be pronounced in several different ways. This allows for polysemy among syllables. One unique feature of Chinese languages is that there are no consonantal blends or clusters before or after the nuclear vowel. The Chinese syllabary contains a total of 22 onsets and 37 rimes and only 2 consonants follow the vowel in the rime of a syllable (velar and alveolar nasal consonants). Homophonic syllables are prevalent in Chinese and these are distinguished by supra-segmental changes in tone that occur in the vowel. Tones change the morphemic content of each syllable. In Putonghua there are four tones and in Cantonese there are eight (at least). There are approximately 400 syllables in spoken Putonghua. However, because syllables can be marked by changes in tone there are 1200 "functional" syllables, each with a different meaning.

All Chinese languages use a non-alphabetic script. A non-alphabetic script is a relatively arbitrary system for mapping orthography to phonology. All Chinese characters are composed of strokes formed into components that are written together into a square shape to form a single character. The traditional script contains over 40,000 characters. However the modern reader needs to learn only the most common 3000 characters to become literate. All characters represent one morpheme in Chinese. This makes the script *morphographic* meaning that the smallest pronounceable unit in a character is associated with a monosyllable. The writing system has also been called logographic. This also means that each written form is associated with a morpheme in the spoken language. This contrasts with letters in an alphabet that do not represent meaning. Ancient Chinese characters were pictographic because the written character portrayed the form of the object that it symbolised. So for example, the character for horse 马 "ma" suggests to some [36,44] an abstract figure galloping across a page. However, few of these are in use today.

There are four different types of character in modern use: (1) pictographic characters that represent a specific object e.g., 口 kou meaning mouth (2) indicative characters that represent abstract meanings that cannot be easily sketched e.g. 本 ben which means 'base' and is derived from 木 mu meaning 'tree'; (3) associative characters that combine existing characters to produce a new meaning e.g. 尘 chen which means 'dust' and is derived from 小 xiao meaning 'small' and 土 tu meaning 'earth'); and (4) phonetic-compound characters that are constructed from a meaning component called the semantic radical and a pronunciation component called the phonetic component e.g. 狐 hu meaning 'fox' which contains a semantic radical denoting animal on the left and a phonetic component pronounced "hu" on the right.

Approximately 80% of characters are compounds. An important point to note is that the phonetic component of a compound is itself often a character (and thus represents a syllable). For example the pronunciation of the character 清 qing is given by the component on the right side of the character. This component can therefore provide information about the pronunciation of the whole character. Similarly, the semantic radical component can often give the reader a clue to the category membership of a character (e.g. an animal) although the semantic radical component is not always a reliable cue for interpreting the meaning of every character.

In a similar way the phonetic information in a compound is also often not a reliable guide to its pronunciation. A regular character contains a phonetic component that is congruent with the pronunciation of the whole character. For example the regular character 坪 pronounced "ping(2)" which is congruent with its phonetic component 平 also pronounced "ping(2)". However, most compounds (>70%) are irregular. This is because there is an unpredictable correspondence between components and the pronunciation of the whole character. For example, the irregular character 秤 pronounced "cheng(4)" is different to the pronunciation of the component 平 pronounced "ping(2)". Furthermore, unlike alphabetic scripts where it is possible to read aloud many words by decoding constituent letters, it is not possible to read aloud a compound by decoding the component parts. This point can be illustrated by considering two facts about Chinese orthography. First, phonetic radicals can be positioned to the left or to the right (or the top or the bottom) of a character. For example, the phonetic radical 其 "qi" is on the right hand side for the character 棋 and means 'chess', but it is on the left hand side for the character 期 which means 'a period of time'. Second, character components can act as both the phonetic radical and the semantic radical in different words. For example, the character 木 *mu* which means 'wood' is a semantic radical in over 1500 Chinese characters, including 棋 qi, however, it is also the phonetic in the character 沐 *mu* which means 'to wash'. This means that it is impossible to know which component in a compound character is the phonetic (or which is semantic) prior to lexical access or from orthographic information alone. To read a character correctly, the reader must know the pronunciation of the character as a whole. In other words reading in Chinese is always a lexical-semantic event [5]. Damage to lexical-semantic processes should therefore result in acquired reading and writing disorders. We describe the characteristics of these disorders in detail below with a focus on reports of acquired surface dyslexia in Chinese.

## 3. Acquired dyslexia in Chinese

Yin [42] and Yin and Butterworth [43] were the first to report Chinese (Putonghua) speakers with a selective impairment to the reading of irregular characters. Their patients – all of whom had cerebrovascular disease – tended to make 'regularization' or more precisely Legitimate Alternative Reading of Component or LARC errors when they read irregular characters aloud [40]. A LARC error in Chinese is an incorrect pronunciation of an irregular character appropriate to other characters containing the same component (cf. [25]). So for example, if the character 秤 is read aloud as "ping(2)" this would be a LARC error. Yin and Butterworth [43] reported an association between semantic impairment and the production of LARC errors when reading irregular characters suggesting that lexical-semantic knowledge might have an impact on the ability to read irregular words in Chinese (as in other scripts [9,21,25,26]). This makes intuitive sense given that the relationship between orthography and phonology is arbitrary for the majority of characters so that, unlike alphabetic scripts, knowing the names of character components will not automatically afford the correct pronunciation of the character.

Weekes and Chen [40] also reported a Chinese speaker with surface dyslexia. Following the results of Yin and Butterworth, they formulated a hypothesis about their patient's reading based on reports of an association between surface dyslexia and lexical-semantic impairment in Chinese and other languages [7,25]. Weekes and Chen [40] reasoned that correct reading of irregular, low frequency, abstract characters requires support from semantic memory and LARC errors are the result of loss of semantic support from the lexical-semantic pathway used to read in Chinese (also [41]). This hypothesis was motivated by theoretical accounts of reading in English which assume knowledge of word meaning – accessed via a lexical-semantic pathway – inhibits LARC errors produced when reading words that have unpredictable components [23,29].

Weekes and Chen defined character regularity according to the *predictability* of character components. If the name of any character component e.g., 青 "qing(1)" had the same name as the character as a whole, then the whole character was called regular e.g., 清 "qing(1)" which means clean. If no character component had the same name as the character then the character was called irregular e.g., 猜 "cai(1)" which means to guess. Therefore regularity referred to the agreement between the pronunciation of a character and its components. Note however that word regularity in English refers to whether a word conforms to grapheme to phoneme correspondence (GPC) rules, and as Chinese does not contain GPC rules, regularity in Chinese is not equivalent to English. Thus, predictable (regular) and unpredictable (irregular) are preferable terms in Chinese.

Weekes and Chen found that their patient LJG's reading of unpredictable characters was worse than predictable characters but only if unpredictable characters were abstract low in word frequency. LJG produced LARC errors e.g. 猜 "cai(1)" would be read as "qing(1)". This is comparable – though not identical – to the regularisation errors reported among patients with surface dyslexia in English. Weekes and Chen characterised LJG as surface dyslexic based on the effects of character predictability on his reading in Chinese. This is compatible to reports of surface alexia in Japanese which uses a logographic script called Kanji [25].

Although we were the first to report surface dyslexia in Chinese Coltheart [4,5] was in fact the first to argue that surface dyslexia could be observed in Chinese speakers. However he did not report patient data and did not focus on effects of regularity (or predictability) on reading. Instead, Coltheart argued that surface dyslexia in Chinese would be characterised by homophone confusions in the comprehension of printed characters. He reasoned that homophone confusions are a consequence of damage to a *lexical* reading pathway which means that access to word meanings from print is achieved indirectly via phonological representations. Coltheart drew an analogy between reading in Chinese and reading of irregular homophonic words e.g., *bury* in English by reasoning that in order to understand the correct meaning of *bury* and not to mistake it for fruit on a tree, it is necessary to derive the correct pronunciation via direct access to the lexicon i.e. from print to meaning. Some patients with surface dyslexia in English do confuse irregularly spelled homophones in reading comprehension [4,5,37]. Hence, one question of theoretical interest is whether homophone confusions in reading comprehension will be observed in Chinese speaking patients who have surface dyslexia.

When Coltheart first theorised about surface dyslexia in Chinese, the question of whether indirect access to word meaning was possible in Chinese was moot. However, we now know that Chinese readers can retrieve phonological representations from print without access to semantic memory. We know this because Weekes, Chen and Yin [39] described an anomic patient called YQS who displayed intact reading of high imageability characters (nouns) including the printed names of objects than she could not name from pictures. Weekes et al. [39] called this *anomia without dyslexia* and interpreted this pattern of aphasia according to the framework depicted in Fig. 1. This framework assumes that reading in Chinese can be preserved despite impaired confrontation naming and reduced category fluency in anomia (that results in a disconnection between the semantic and phonological representations depicted in the framework). The pattern of poor picture naming with superior reading of characters has subsequently been replicated by Weekes and Chen [40] in another Putonghua speaking patient and also by Law and Or [16] who reported a Cantonese speaking patient CML with a similar pattern of spared oral reading together with impaired naming performance (see also [14,15]; Law and colleagues this volume).

If it is true that surface dyslexia in Chinese is associated with indirect access to character meanings from print (as argued by Coltheart), then the comprehension of homophonic characters should be impaired as in patients who have surface dyslexia in other languages. Furthermore, if homophone confusions are due to problems with mappings between orthography and meaning then visually similar homophones might cause more interference on comprehension tasks. This hypothesis was tested with LJG in two tasks. In each task, twenty printed monosyllabic object names were selected according to the criterion that LJG could read aloud the character correctly. All items in both tasks are shown in Table 1. In Task 1, LJG was presented with a picture of an object surrounded by four characters (randomly on each trial). The printed name of the object (target) and three characters (distractors): one homophonic but visually dissimilar character and two non-homophonic characters one that was visually similar and the other visually dissimilar to the target. LJG was asked to

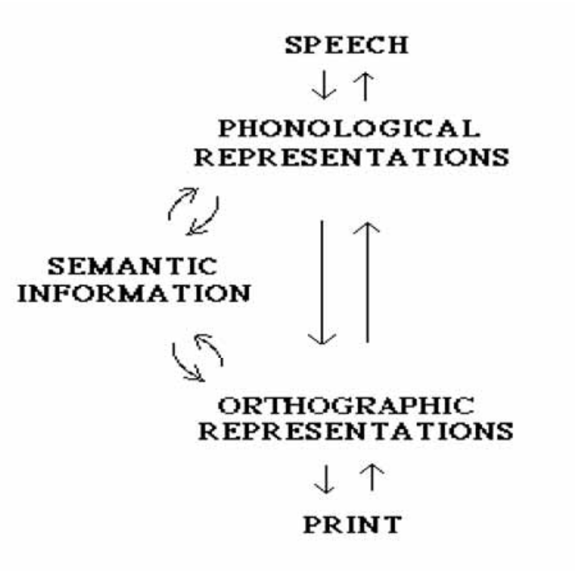

Fig. 1. Functional model describing reading and writing in Chinese with a lexical-semantic pathway (left of figure) and a nonsemantic pathway (right of figure).

match the character to the picture with the instruction not to read any character aloud. He performed this task with 100% accuracy. In Task 2, LJG was presented with twenty objects surrounded by five characters (randomly for each trial): the printed name of the object (target) and four characters as distractors; one homophonic and visually similar character; one homophonic but visually dissimilar character; and two non-homophonic characters – one visually similar and the other visually dissimilar to the target. Phonologically similar and visually similar characters were included in this task to test the possibility that phonological and visual interference may have additive effects on performance. His performance was 15/20 (75%) correct. Errors were always the incorrect selection of homophonic and visually similar distractors. In a test of homophone comprehension, LJG was presented with the stimuli from Task 1 and asked to point (silently) to the character with the same name as the target. He performed with100% accuracy on this task.

The results from LJG show that homophone confusions in reading comprehension can be observed in Chinese surface dyslexia as predicted by Coltheart [5]. However errors are limited to visually similar homophones. This suggests that homophone confusions are due to impairment to the mappings between orthography and word meaning. LJG could retrieve the meaning of all characters in Task 1 and he did not select visually similar non-homophonous characters on either

Table 1
Items used in tests of reading comprehension

Task 1

| Picture | Target | P+V- | P-V+ | P-V- |
|---|---|---|---|---|
| | 嘴 | 最 | 嘀 | 伸 |
| | 锚 | 毛 | 锡 | 途 |
| | 虾 | 夏 | 独 | 点 |
| | 鸟 | 脲 | 鸪 | 肌 |
| | 鱼 | 揄 | 笛 | 末 |
| | 腿 | 颓 | 膀 | 捎 |
| | 马 | 嘛 | 驴 | 僵 |
| | 熊 | 雄 | 烈 | 申 |
| | 手 | 寿 | 撑 | 旦 |
| | 鹿 | 卤 | 庞 | [illegible] |
| | 旗 | 起 | 痒 | 舞 |
| | 门 | 懑 | 闪 | 方 |
| | 锁 | 所 | 缘 | 愁 |
| | 伞 | 三 | 全 | 了 |
| | 球 | 丘 | 浆 | 价 |
| | 书 | 术 | 十 | 公 |
| | 钟 | 重 | 针 | 网 |
| | 驴 | 率 | 吗 | 茶 |
| | 象 | 项 | 竽 | 查 |

Task 2

| Picture | Target | P-V+ | P+V- | P+V+ | P-V- |
|---|---|---|---|---|---|
| | 床 | 庆 | 创 | 疮 | 翔 |
| | 船 | 铅 | 串 | 舡 | 春 |
| | 碗 | 碱 | 顽 | 婉 | 葬 |
| | 糖 | 粹 | 躺 | 搪 | 新 |
| | 云 | 亏 | 允 | 运 | 半 |
| | 狗 | 拘 | 垢 | 佝 | 帛 |
| | 鼓 | 彭 | 辜 | 故 | 零 |
| | 花 | 符 | 划 | 化 | 应 |
| | 脚 | 卿 | 焦 | 胶 | 离 |
| | 枪 | 枸 | 墙 | 抢 | 昏 |
| | 针 | 叶 | 枕 | 镇 | 临 |
| | 鹰 | 腐 | 影 | 膺 | 壁 |
| | 梨 | 染 | 理 | 犁 | 善 |
| | 猪 | 赌 | 柱 | 箸 | 晨 |
| | 锯 | 镇 | 菊 | 据 | 崩 |
| | 鞋 | 蛙 | 谢 | 斜 | 墨 |
| | 蛇 | 舵 | 社 | 射 | 寄 |
| | 剑 | 敛 | 芭 | 检 | 另 |
| | 树 | 椒 | 殊 | 柜 | 都 |

task. Thus he was able to use orthographic information to identify the meaning of homophonic characters. This means that if access to semantics from orthography is only partially damaged, surface dyslexia can be observed in Chinese. Moreover, visual similarity did not interfere with LJG's ability to retrieve phonology from print as shown by his performance on the homophone matching tasks. According to Coltheart [5], homophone confusions in comprehension show that activation of meaning from orthography is achieved via phonological activation that is derived *indirectly*. However according to Fig. 1, surface dyslexia may arise from damage to semantics or the mappings between orthography and semantics (O->S) or semantics and phonology (S->P).

The observation that LJG makes homophone confusions informs theorists and clinicians about the functional architecture of the reading system as well as cognitive processes used in normal and impaired reading in Chinese. Firstly, LJG's performance is compatible with the now widely held view that Chinese readers can activate meaning via phonological mediation [17, 28,34]. Second, LJG's reading errors suggest that the mappings between orthography and semantics may be important for inhibiting (incorrect) access to semantics via phonology. Thus, damage to the mappings between orthography and semantic representation of words has a substantial effect on the comprehension of print in Chinese – although reading comprehension may be impaired in surface dyslexia in English [37] and also in Spanish (see Ferreres and colleagues this volume).

## 4. Acquired dysgraphia in Chinese

Law and Or [16] reported that their patient CML displayed superior writing to dictation compared with her written picture naming a pattern that was also mirrored in better performance on tests of oral reading compared to picture naming (as for patient YQS (see [39])). They argued that CML's impairments showed orthographic output is produced by an isolated nonsemantic pathway that is normally available for writing characters in Chinese. Reich, Chou and Patterson [31] reported a Cantonese speaker with acquired dysgraphia who showed a similar pattern. TUA made few errors writing high-frequency words to dictation, but had error rates of 60–70% with lower-frequency homophonic target words. Most errors were phonologically related to the target. TUA's deficit in writing was accompanied by good definition of the correct lower-frequency homophonic alternative. Reich et al. argued that TUA was impaired at writing via the lexical-semantic pathway. Law [13] reported a Cantonese speaker whose errors in writing-to-dictation and written naming were phonologically plausible errors that were homophonous or differed in tone from the target. Law found that non-character responses, particularly involving substitution of constituent(s) maintained spatial configuration of the target and proposed that the orthographic representations of characters contain information on the identity of components and structural information as a template of the organisation of the character or specification for position of occurrence for each constituent within the character. The homophone errors in writing in these studies lend support to the notion of two pathways for writing in Chinese [16]. When isolated, the nonsemantic pathway produces phonologically plausible responses (homophonic characters). This is comparable though not identical to the pattern of surface dysgraphic errors in alphabetic languages [37]. Of course, the writing errors in Chinese dysgraphia cannot be equivalent to the writing errors in other languages because of the unique spatial characteristics of the script.

## 5. A functional account of acquired dyslexia and dysgraphia in Chinese

Weekes et al. [39] argued that normal oral reading in Chinese can proceed via at least two pathways: *the lexical semantic pathway* that supports reading for meaning; and a *nonsemantic pathway* that connects the representations of orthography i.e. strokes, radicals and characters to phonological representations i.e. syllables, rimes and tones. Note that this cognitive framework can also explain writing to dictation via access from semantics to orthography and a nonsemantic pathway [16] if mappings between orthography and phonology are assumed to be bidirectional.

A character will be processed in both pathways during the course of normal reading and writing. For reading, an orthographic representation will activate lexical representations related by meaning via the lexical semantic pathway or by sound via the nonsemantic pathway. Many representations could be produced as a response. The same process is assumed to work in reverse for writing to dictation. However, errors are not usually observed in normal Chinese reading and writing (though see [22]). This is because input from the nonsemantic pathway can inhibit the semantically related (incorrect) reading and writing responses generated by the lexical-semantic pathway and input from the lexical-semantic pathway can inhibit the phonologically plausible i.e., LARC, homophonic and tonal reading and writing responses generated by the nonsemantic pathway. If either pathway is unavailable (due to brain damage), selective reading and writing errors will be observed. Our reasoning is compatible with Hillis and Caramazza's summation hypothesis [11], which assumes that normal reading (and writing) depends on the summation of pooled activation at the level of phonological output from multiple reading pathways.

Damage to the nonsemantic pathway in Chinese will result in *semantic dyslexia* that is characterised by semantic errors in reading such as those reported in patients with deep dyslexia in other languages (e.g. see Davies and Cuetos this volume). For example, Law [14] describes the spoken word output of a Cantonese-speaking patient who had intact comprehension but produced semantic errors in reading (and naming) and also produced no LARC errors in oral reading (see also Yin and Butterworth [43] for reports of semantic reading errors in Putonghua speakers). Law [14] reasoned that a deficit to processes in the lexical semantic pathway results in semantic errors when accompanied by a damaged nonsemantic pathway (see Raman and Weekes and Davies and Cuetos this volume for similar accounts of deep dyslexia and dysgraphia). Law [15] also reported LKK who made more semantic errors in picture naming than oral reading suggesting semantic errors are inhibited if there is input from the nonsemantic reading pathway.

Damage to the lexical semantic pathway results in LARC errors. In reading, the normal system is able to inhibit competing and more common pronunciations of character components at the level of phonological output. This inhibition will prevent LARC errors when reading aloud. The reading system will settle on the correct but atypical pronunciation of an irregular word via input from semantic memory. However without semantic support, more common pronunciations of components might dominate computation of phonology from orthography. Thus, impairment to semantic memory processing is associated with surface dyslexia in Chinese (see also papers Patterson and colleagues for reports in English and Japanese [9,10, 25]). According to Law and Or (2001), the nonsemantic pathway if isolated produces writing errors that are phonologically plausible (as in surface dysgraphia in English). This is because orthographic output from an isolated nonsemantic pathway will be homophonic with the target [16]. Therefore the semantic support hypothesis [23,29] can explain the production of LARC errors in alphabetic and non-alphabetic scripts [9,25, 40] as well as production of homophone errors in writing in both types of script [13,16] i.e., without input from the lexical-semantic pathway there will be no inhibition over production of phonological reading and writing errors (see also Hillis & Caramazza [11]).

## 6. Summary

We have illustrated the similarities between acquired dyslexia and dysgraphia in Chinese and alphabetic languages with a focus on phonological reading and writing errors reported in surface dyslexia and dysgraphia. However, this does not imply that disorders of reading and writing should be viewed as universal across scripts see [1,8,21,30]. In fact the patterns of acquired dyslexia and dysgraphia observed in Chinese cannot be identical to the patterns observed in alphabetic languages. This is because certain properties of the Chinese script do not allow direct comparisons between the disorders. For example, nonlexical stimuli in alphabetic scripts e.g. zint simply cannot be constructed in Chinese. Thus, it will not be possible to replicate the pattern of preserved nonword processing in surface dyslexia and dysgraphia observed in English and Spanish [5].

Instead patterns of acquired dyslexia and dysgraphia in Chinese will be determined by the properties of the language environment. This point can be illustrated by the intriguing reports of tonal dyslexia in Chinese. Law and Or [16] reported that CML produced correct monosyllables on reading tasks but assigned the wrong tonal stress to the syllable. Eng and Obler [8] reported this phenomenon in a biscriptal Cantonese-English speaker and Luo and Weekes [18] reported this phenomenon in a Putonghua speaker. CML also produced tonal errors and homophone errors (same syllable and tone) when writing from dictation and writing the names of pictures (see also [13]). Tonal errors in reading and writing are informative about phonological representations in the Chinese lexicon. One view of phonological representations of Chinese words is that they have a nonlinear structure containing separate syllabic, segmental (onset and rime) and supra-segmental layers. For Law and Or (2001), tonal reading and writing errors result from impairment to the tonal tier or association between tonal and segmental tiers which leads to a dissociation between segmental and suprasegmental units, thus revealing the structure of the phonological lexicon.

A key question in neurology is how different languages are processed in the brain. The Chinese language environment is unique and, in the domain of reading and writing, logographic writing systems have evolved in a very different way to alphabetic scripts. We have approached the problem of acquired dyslexia and dysgraphia in Chinese by testing predictions derived from a cognitive framework that was developed to explain oral reading and writing in Chinese. This is the cognitive neuropsychological approach to understanding reading and writing that has been used to explain dyslexia and dysgraphia in a variety of languages [1–3, 7,9–11,19–21,23–27,30,37,38].

It has long been recognised that damage to the reading/writing system can produce a particular type of error for different reasons (e.g. [32]). This logic can be applied to explaining the symptoms of acquired dyslexia and dysgraphia across scripts. Our approach to understanding reading and writing has resonance with more sophisticated computational models of oral reading in Chinese [28] and recent brain imaging studies [33,35]. The next challenge for aphasiologists, cognitive neuropsychologists and neurolinguists is to specify how common cognitive processes that have evolved in the brain have adapted to the properties of different language environments.

## Acknowledgements

This research was supported by Grants from: the Australian Research Council, the Royal Society and the Research Grants Council of the Hong Kong Government (HKU7275/03H).
